# Ferrocenyl Phosphorhydrazone Dendrimers Synthesis, and Electrochemical and Catalytic Properties

**DOI:** 10.3390/molecules25030447

**Published:** 2020-01-21

**Authors:** Cédric-Olivier Turrin, Eric Manoury, Anne-Marie Caminade

**Affiliations:** 1 Laboratoire de Chimie de Coordination du CNRS, 205 Route de Narbonne, BP 44099, 31077 Toulouse CEDEX 4, France; cedric-olivier.turrin@lcc-toulouse.fr (C.-O.T.); eric.manoury@lcc-toulouse.fr (E.M.); 2 LCC-CNRS, Université de Toulouse, CNRS, Toulouse, France

**Keywords:** ferrocene, dendrimer, catalysis, electrochemistry

## Abstract

The discovery of ferrocene is often associated with the rapid growth of organometallic chemistry. Dendrimers are highly branched macromolecules that can be functionalized at will at all levels of their structure. The functionalization of dendrimers with ferrocene derivatives can be carried out easily as terminal functions on the surface, but also at the core, or at one or several layers inside the structure. This review will focus on phosphorhydrazone dendrimers functionalized with ferrocene derivatives, on the surface, at the core, at all layers or within a single layer inside the structure. The first part will describe the synthesis; the second part will concern the electrochemical properties; and the last part will give several examples concerning catalysis, with complexes of ferrocenyl phosphines used as terminal functions of dendrimers.

## 1. Introduction

**Figure 1:**
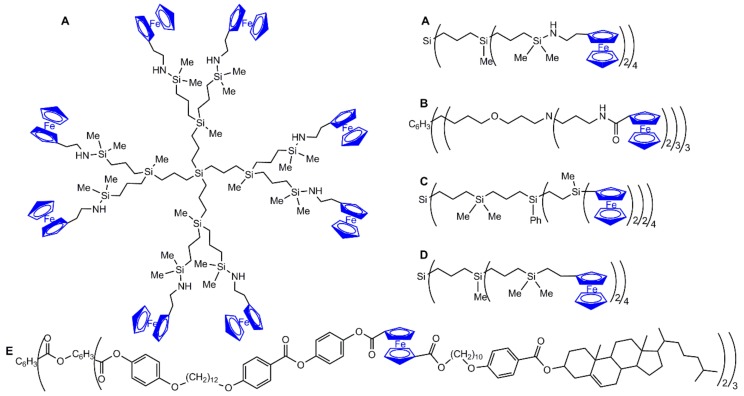
Early examples of ferrocenyl dendrimers. Dendrimer **A** is drawn two times: the full structure is on the left and the linear structure is on the right. The other dendrimers (**B** to **E**) are drawn linearly.

Thousands of publications concern ferrocene derivatives, and their discovery represented a turning point in organometallic chemistry, as the leading member of the metallocene family. Ferrocene consists of two cyclopentadienyl rings bound on opposite sides of a central Fe^2+^ atom. It has been shown to have excellent thermal and photochemical stability, and to undergo a facile and reversible one-electron oxidation to ferrocenium cation. The functionalization of one or both cyclopentadienyl units has led to their grafting to numerous types of molecules, and to polymers and to dendrimers. Dendrimers are a very special class of polymers, constituted of repetitive branching units but not synthesized by polymerization reactions. Their step-by-step synthesis ensures a perfectly defined and highly reproducible structure [1]. Each layer constitutes a generation. The grafting of ferrocenes to dendrimers was carried out early on the periphery, in particular for carbosilane dendrimers [2]. This dendrimer is represented in two different ways in Figure 1A: either the full structure in the left part or in a linear way, with parentheses after each branching point (each generation) in the right part. All the other ferrocenyl dendrimers are represented in this linear way. Diverse properties of ferrocenyl dendrimers have been also discovered early, such as redox sensors for the recognition of small inorganic anions with amido ferrocene dendrimers (Figure 1B) [3], as guests for the inclusion of *β*-cyclodextrin with polyamine ferrocene dendrimers (Figure 1C) [4], as mediators in amperometric biosensors with carbosilane ferrocene dendrimers (Figure 1D) [5], or as liquid crystals with ferrocene-based cholesterol dendrimers (Figure 1E). In this last case, the ferrocene is not on the top of the dendrimer surface but is linked by a long alkyl chain to cholesteryl units [6]. Several hundreds of papers concerning diverse types of ferrocenyl dendrimers have since been published.

**Figure 2:**
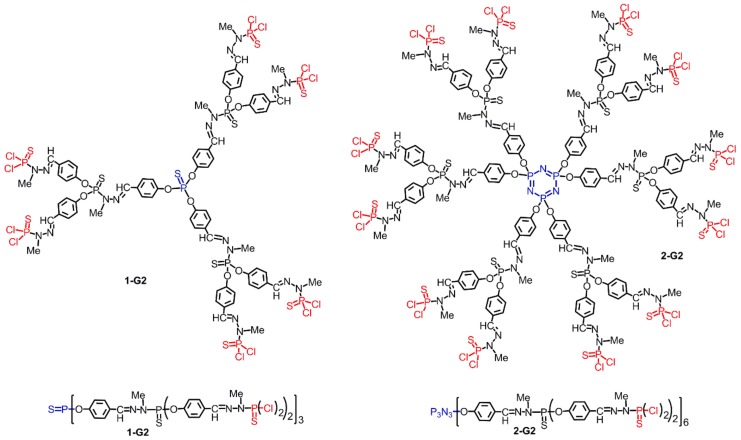
Structure of second generation phosphorhydrazone dendrimers. Left: built from P(S)Cl_3_ as core; right: built from N_3_P_3_Cl_6_ as core. Upper part: full structure; lower part: linear structures with parentheses to draw the same dendrimers.

Among the diverse types of dendrimers, phosphorhydrazone dendrimers, which possess one phosphorus atom at each branching point [7], occupy a special place among “inorganic” dendrimers [8] thanks to their easy and versatile functionalization, in particular on their periphery [9], and to the numerous properties that have been already demonstrated with them. For instance, they have been used as catalysts [10,11,12] for materials [13], in biology [14,15,16], and in nanomedicine [17]. Figure 2 displays the chemical structure of phosphorhydrazone dendrimers of generation 2, built either from P(S)Cl_3_ (**1-G2**) or cyclotriphosphazene N_3_P_3_Cl_6_ (**2-G2**) [18]. Their synthesis necessitates the repetition of two quantitative reactions: the reaction of hydroxybenzaldehyde with P-Cl functions and the condensation of the aldehydes with phosphorhydrazide H_2_NNMeP(S)Cl_2_.

In this review, we will focus on the synthesis and electrochemical and catalytic properties of ferrocenyl phosphorhydrazone dendrimers.

## 2. Synthesis of Ferrocenyl Phosphorhydrazone Dendrimers

Depending on the desired location of the ferrocenes in the structure of phosphorhydrazone dendrimers, the ferrocenes should be functionalized differently. For the grafting to the surface, the ferrocene should bear a phenol group, suitable to react in substitution reactions with the P(S)Cl_2_ terminal groups of the dendrimers. To be used as core, the ferrocene should have two aldehyde functions, from which the synthesis of the dendrimer will be carried out. To be introduced in the branches, at all layers or within a single layer, the ferrocene should replace hydroxybenzaldehyde; thus, it should bear both a phenol and an aldehyde. The synthesis part will be presented depending on the location of the ferrocenes in the structure of the phosphorhydrazone dendrimers.

### 2.1. Ferrocenes on the Periphery of Dendrimers

**Figure 3:**
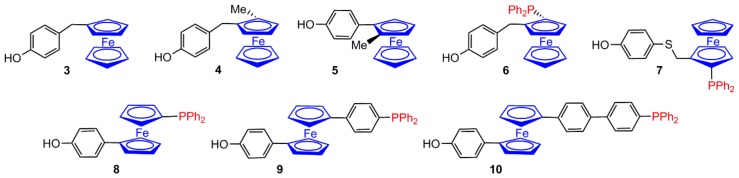
Ferrocenylphenols that have been linked to the surface of phosphorhydrazone dendrimers.

The functionalization of ferrocene was carried out thanks to several efficient syntheses [19,20,21,22]. All the ferrocenes that have been linked to the surface of phosphorhydazone dendrimers are functionalized by a phenol, and possibly by another function, in particular a phosphine. The different types of these ferrocenes are shown in Figure 3. The ferrocene derivative **3** was linked to the surface of dendrimers of type **1-Gn** (P(S)Cl_3_ as core), up to generation 9 (**3-G9**, 1536 ferrocenyl groups) [23]. The chiral ferrocene derivative **4** (planar chirality) was linked to the surface of the same type of dendrimers, up to generation 3 (**4-G3**, 24 ferrocenyl groups), whereas the chiral ferrocene derivative **5** was linked to the eleventh generation (**5-G11**, 6144 ferrocenyl groups) [24]. The chiral phosphino ferrocene **6** was also linked to the surface of the same dendrimers, up to generation 9 (**6-G9**, 1536 ferrocenyl groups) [24]. The chiral phosphino ferrocene **7** was linked to the surface of dendrimers of type **2-Gn** (N_3_P_3_Cl_6_ as core), up to generation 4 (**7-G4**, 96 ferrocenyl groups) [25]. The phosphino ferrocenes **8**, **9**, and **10** have been linked to the surface dendrimers **2-Gn**, up to generation 3 (**8-G3**, **9-G3**, and **10-G3**, respectively, 48 ferrocenyl groups in all cases) [26].

**Figure 4:**
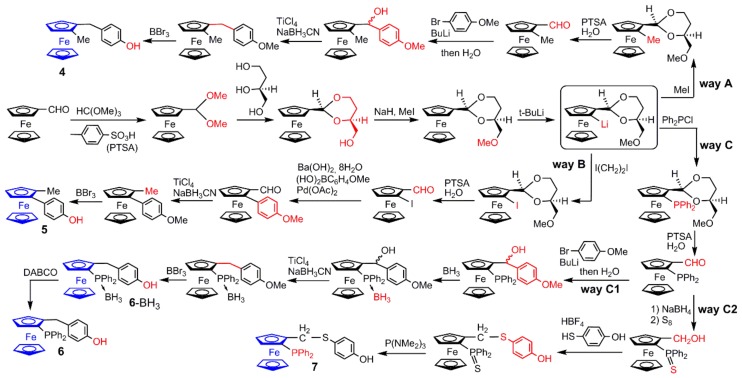
Synthesis of ferrocenes **4**, **5**, **6**, and **7**, starting from the common precursor ferrocene carboxaldehyde.

The synthesis of such functionalized ferrocenes is not trivial, and necessitates in all cases several steps from commercially available ferrocenes. Ferrocenes having a planar chirality (ferrocenes **4**, **5**, **6**, and **7**) are all synthesized starting from a common precursor, ferrocene carboxaldehyde, as illustrated in Figure 4. The first steps are also identical for all compounds. The aldehyde is quantitatively converted to the acetal using neat trimethylorthoformate. Trans acetalization was carried out with (*S*)-(-)-1,2,4-butanetriol, to provide another acetal, having the *cis*-1,3-dioxane structure. Methylation of the hydroxyl group is then carried out using NaH and MeI. Ortho-deprotonation was carried out using *t*-BuLi. The lithium derivative is the last common precursor, which is reacted with diverse electrophiles [27]. The electrophile used is MeI for the synthesis of ferrocene **4** (way A), ICH_2_CH_2_I for ferrocene **5** (way B), and Ph_2_PCl for ferrocenes **6** and **7** (way C) (Figure 4). The deprotection of the acetal to recover the aldehyde is carried out in all cases using *p*-toluene sulfonic acid (PTSA) and water.

The next step in way A is the reaction of 4-lithioanisole obtained by transmetalation of 4-bromoanisole by n-butylithium on the aldehyde, followed by deoxygenation with sodium cyanoborohydride in presence of titanium tetrachloride. Finally, the methoxy group is transformed to hydroxyl group by reaction with boron bromide, to provide the ferrocene **4**, after 9 steps from ferrocene carboxaldehyde [24]. The ferrocene derivative functionalized with both an aldehyde and an iodine (way B) is reacted in a Suzuki coupling reaction with 4-methoxyphenylboronic acid to yield enantiomerically pure 4-methoxyphenyl group on the 2 position of ferrocenecarboxaldehyde. The aldehyde is then converted to a methyl group using sodium cyanoborohydride in presence of titanium tetrachloride Finally, the methoxy group is deprotected to provide compound **5** after 9 steps, as was obtained ferrocene **4** [24]. The (S)-2-(diphenylphosphino) ferrocene carboxaldehyde obtained in way C is the precursor of both ferrocenes **6** (way C1) and **7** (way C2). In way C1, 4-lithioanisole is first reacted on the aldehyde; then, the phosphine is protected with BH_3_. Reactions with sodium cyanoborohydride in presence of titanium tetrachloride and then with boron bromide are carried out as in way A, to provide compound **6**-BH_3_. The phosphine is finally deprotected using DABCO (1,4-diazabicyclo[2.2.2]octane) to provide the ferrocene **6** after 11 steps [24]. In way C2, the aldehyde is reduced to alcohol and the phosphine is oxidized to thiophosphine using sulfur. In a second step, 4-hydroxythiophenol is reacted on the alcohol, providing directly a phenol. The last step is the reduction of the thiophosphine with tris (dimethylamino)phosphine, providing the ferrocene **7** in 9 steps (Figure 4) [25].

**Figure 5:**
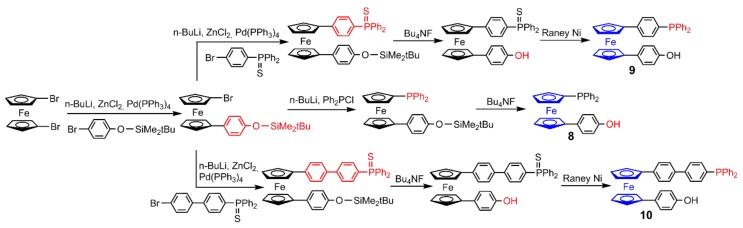
Synthesis of ferrocenes **8**, **9**, and **10**, starting from the common precursor 1,1’-dibromoferrocene.

The ferrocenes functionalized on both cyclopentadienyl rings (compounds **8**, **9**, and **10**) are easier to synthesize, as shown in Figure 5. The common precursor in these cases is 1,1’-dibromoferrocene, on which one bromine is treated with 4-bromoanisole in a Negishi cross-coupling reaction. The direct grafting of diphenylphosphine on the other cyclopentadienyl ring is carried out by lithiating the ferrocene, then quenching with diphenylchlorophosphine. The deprotection of the phenol using tetrabutylammonium fluoride provides the ferrocene **8** after only three steps (Figure 5) [26]. A slightly different procedure is used for introducing a linker between the ferrocene and the phosphine (compounds **9** and **10**). The lithiated ferrocene in presence of ZnCl_2_ and [Pd(PPh_3_)_4_] is treated with either 4-bromophenyl-diphenylphosphane sulfide or 4’-bromobiphenyl diphenylphosphane sulfide. The corresponding phenol is obtained as previously using tetrabutylammonium fluoride. The last step is the deprotection of the thiophosphine, using Raney nickel, to provide ferrocenes **9** or **10** in only four steps, depending on the linker used (Figure 5) [26].

### 2.2. Ferrocene at the Core of Dendrimers

**Figure 6:**
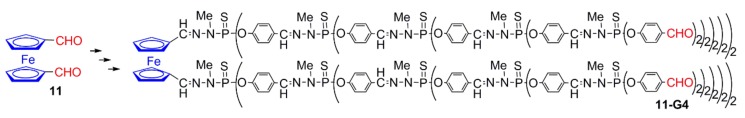
1,1’-ferrocene dicarboxaldehyde used as core of dendrimer **11-G4**.

Ferrocene derivatives usable as core should have at least two identical functions. The commercially available 1,1’-ferrocene dicarboxaldehyde **11** was used as core to grow the dendrimer up to generation 4 (**11-G4**, 64 aldehyde terminal functions) (Figure 6) [23].

### 2.3. Ferrocenes as Branches of Dendrimers at one or Several Layers

**Figure 7:**
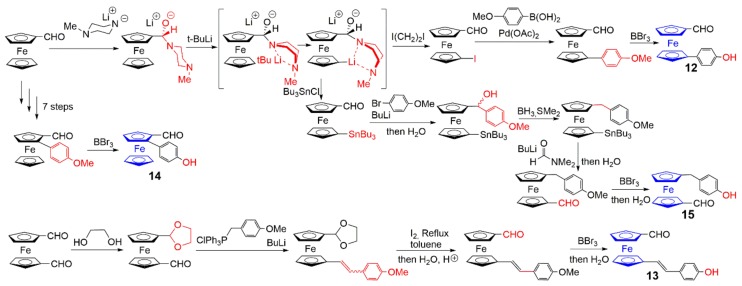
Methods of synthesis of ferrocenes functionalized by both a phenol and an aldehyde, which are suitable to be used as branches inside dendrimers. The seven steps for the synthesis of **14** are shown in Figure 4.

The functionalization of ferrocenes usable as branches of phosphorhydrazone dendrimers necessitates one to have a phenol on one side and an aldehyde on the other side. Several ferrocene derivatives have been synthesized bearing both suitable functions, and have been used either at several layers of small dendrimers (ferrocenes **12** and **13**, Figure 7) or at a single layer inside the structure of large dendrimers (ferrocenes **14** and **15**, Figure 7). The methods of synthesis are relatively similar to those shown in Figure 4. In particular, the ferrocene **14** is obtained by transformation of the methoxy to hydroxyl group in the final step, after seven steps shown in Figure 4 [24]. The main difference between the reactions in Figure 4 and Figure 7 concerns the reaction of ferrocene carboxaldehyde with the lithium salt of *N*-methylpiperazine, which, after additional reaction with *t*-BuLi, generates the lithation of the other cyclopentadienyl ring [19]. Reaction with electrophiles such as ICH_2_CH_2_I or Bu_3_SnCl are then carried out. The iodoferrocene is first reacted in a Suzuki coupling reaction with 4-methoxyphenylboronic acid, and then the methoxy group is deprotected with BBr_3_ to provide the ferrocene **12** [23]. The stannyl derivative is first reacted on the aldehyde with 4-lithioanisole, and then the alcohol is reduced with BH_3_,SMe_2_. The tributylstannyl group is converted to an aldehyde using butyl lithium, dimethylformamide, and then water. The final step for the synthesis of compound **15** is the cleavage of the methoxy group to provide the phenol, as done previously for compound **12**.

Ferrocene **13** is obtained in a different way, as the starting compound for its synthesis is the 1,1’-ferrocene dicarboxaldehyde. A first aldehyde is protected with ethylene glycol, then the second aldehyde is reacted with 4-methoxybenzyltriphenylphosphonium chloride in the presence of *n*-BuLi to yield a mixture of diastereoisomer (**E**/**Z** = 1) by a Wittig reaction. Reaction with iodine in refluxing toluene followed by an acidic hydrolysis provides a complete conversion to the E-isomer of the corresponding aldehyde. The last step to get ferrocene **13** is the deprotection of the methoxy group to hydroxyl, as carried out for many other ferrocenes shown in this review.

#### 2.3.1. Ferrocenes at all Layers of Dendrimers

**Figure 8:**
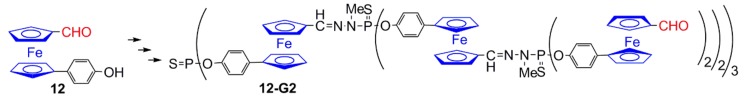
Bifunctional ferrocene **12** used at all layers of dendrimer **12-G2**.

The bifunctional ferrocene **12** was first reacted with the P(S)Cl_3_ core; then, it was used alternately with the phosphorhydrazide H_2_NNMeP(S)Cl_2_, up to the second generation **12-G2** (21 ferrocene derivatives and 12 aldehyde terminal groups) (Figure 8) [23].

**Figure 9:**
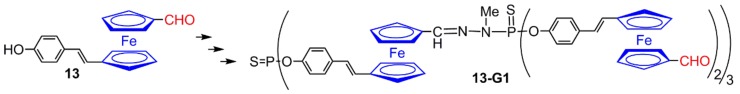
Bifunctional ferrocene **13** used at all layers of dendrimer **13-G1**.

The same process was applied to the other bifunctional ferrocene **13**, and was carried out up to the first generation **13-G1** (9 ferrocene derivatives and 6 aldehyde terminal functions) (Figure 9) [28].

#### 2.3.2. Ferrocenes at a Single Layer Inside Dendrimers

**Figure 10:**
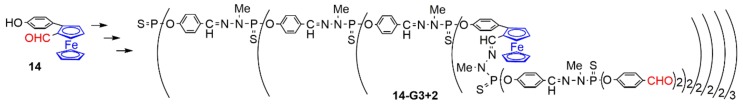
Bifunctional ferrocene **14** used at a single layer of dendrimer, and dendrimer **14-G3+2**.

The chiral ferrocene **14** was introduced at a single layer in phosphorhydrazone dendrimers. It was used first on the surface of dendrimer **1-G3**, providing dendrimer **14-G3** [24], then the growing of the dendritic shell was carried out to obtain **14-G3+1**, then **14-G3+2**, as illustrated in Figure 10 (24 ferrocenyl groups for all). The same type of experiment was carried out starting from **1-G5** and **1-G9**, providing **14-G5+2** (96 ferrocenyl groups) and **14-G9+2** (1536 ferrocenyl groups), respectively [29].

**Figure 11:**
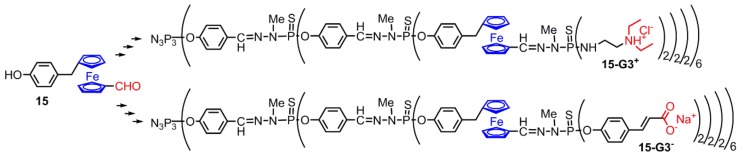
Bifunctional ferrocene **15** used at a single layer of water-soluble dendrimers **15-G3^+^** and **15-G3^−^**.

Other dendrimers bearing a single layer of ferrocenes were also obtained, starting from the dendrimer **2-G2**, onto which 24 ferrocenyl derivatives **15** were grafted. The growing of the dendrimer was continued in order to get water-soluble ferrocenyl dendrimers. Ammonium derivatives have been linked to the surface, providing the positively charged dendrimer **15-G3^+^**, whereas carboxylates have been used to provide the negatively charged dendrimer **15-G3^-^** (Figure 11) [30].

## 3. Electrochemical Properties

Due to the well-known facile and reversible one-electron oxidation of ferrocene to ferrocenium cation, the electrochemical properties of most of the ferrocenyl phosphorhydrazone dendrimers have been studied. For ferrocenes on the periphery of dendrimers, such as the family **3-Gn** (n up to 9), a single oxidation wave at ca. 500 mV is observed, corresponding to a multielectronic transfer of the equivalent and electrochemically independent ferrocenyl terminal groups. The fact that this behavior is still observed for the ninth generation **3-G9** confirms the absence of steric hindrance on the surface of this family of dendrimers. The multiferrocenium salt obtained upon exhaustive electrolysis deposits onto the Pt gauze electrode surface to form a blue-green conducting film, which totally dissolves upon reduction. The theoretical number of transferred electrons is never completed; however, 23/24 of the FeCp_2_ sites are oxidized for **3-G3**, 94/96 for **3-G5**, and 1352/1536 for **3-G9**. This observation must not be systematically correlated to defects on the surface. Indeed, even very simple monoferrocenyl compound gives less than 100% charge recovery [23]. Even for the eleventh generation **5-G11**, which is at the limit of dense packing, 87% of the theoretical number FeCp_2_ sites are oxidized, to be compared with 88% for the fifth generation **5-G5**, showing that there is no influence of the generation on the redox properties [24]. No electrochemical study was attempted for dendrimers having ferrocenyl phosphines as terminal functions, except when the phosphine was complexed with ruthenium (case of compounds **8-Gn**, **9-Gn,** and **10-Gn**, **n** = 1 to 3). In these cases, a fully reversible one-electron redox process was observed for the ferrocenes between 0.42 and 0.55 V. The behavior of the Ru complex depended on the generation and on the linker: the monomer and the first generation **8-G1-Ru** showed an irreversible oxidation of Ru^2+^, whereas the higher generations and dendrimers having the other linkers (**9-Gn-Ru** and **10-Gn-Ru**) displayed a reversible oxidation of Ru^2+^ [26].

The electrochemical response of dendrimers having a single ferrocene at the core (dendrimers **11-Gn**) is highly dependent on the generation. The cyclic voltammograms of the monomer and of the lower generation dendrimers **11-G0** show characteristic quasi reversible single electron oxidation processes with production of soluble and stable cations. The first generation dendrimer **11-G1** exhibited a slightly more irreversible process, which increased for compound **11-G2**, whereas a flat current response was obtained for **11-G3**. The electroactive core of dendrimers presenting sufficient steric hindrance cannot approach the electrode close enough, and so no current could be detected for high generations [23].

**Figure 12:**
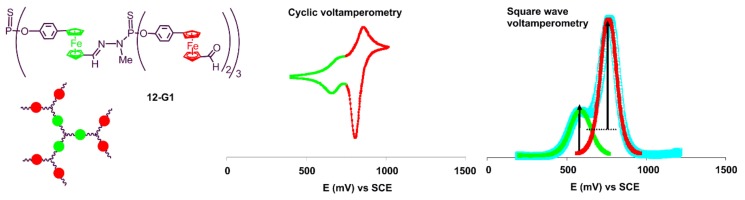
Cyclic voltamperometry and square wave voltamperometry of dendrimer **12-G1** in acetone/THF (1:2), versus standard calomel electrode (SCE).

The case of dendrimers having several layers of ferrocenes, such as the family **12-Gn**, is particularly interesting, as the ferrocenes are in a different environment, depending on their location. Dendrimer **12-G0** presents 3 ferrocenyl moieties, which are equivalent. Dendrimer **12-G1** possessing two layers of ferrocenyl moieties exhibits a first wave at E1/2 = 0.68 V, and a second one at E1/2 = 0.83 V. The presence of two different waves unambiguously proves that both layers behave independently. Bulk electrolysis at controlled potential allowed one to count three electrons (with a 95% oxidation ratio) involved in the first transfer. Further electrolysis of this dendrimer was impossible to perform due to deposition upon the Pt working electrode. Nevertheless, the second wave was assigned to the outermost ferrocenes, comparing the current peak intensities measured by square wave voltammetry technique (Figure 12). Dendrimer **12-G2** has three levels of ferrocenes, but only two waves were observed either in cyclic or square wave voltammetry at E1/2 = 0.66 and 0.80 V. Exhaustive oxidation at 0.70 V allowed for the counting of 10 electrons instead of the nine electrons expected for both the first and the second layers, probably due to a partial oxidation of the third layer resulting from the poor resolution of the second signal [23]. The other dendrimer having two layers of ferrocenes (**13-G1**) behaved similarly to **12-G1** [28].

The dendrimers having a single layer of ferrocenes in their structure produced different results, depending on the degree of “burying” of the ferrocenes. The value of the half-wave potential strongly depends on the direct chemical environment of the ferrocenes. The replacement of the aldehyde of the **14-Gn** family by the hydrazone induced a shift of ca 160 mV toward anodic potentials, whatever the size of the dendrimer considered. By continuing the growing of the dendrimers, the rate of the electronic transfer decreased, and the reversibility of the system decreased when the generation of the dendrimer increased within the same series. These phenomena can be explained by the decreased accessibility of the electrode for the redox centers, which was gradually excluded from the electronic transfers for the **14-Gn** to **14-Gn+2** families. These families of dendrimers being chiral, the chiroptical properties were also investigated. The molar rotation, the [*α*]_mol_ value, indicates a large variation between the **14-Gn** and **14-Gn+1** series and a very small variation between the **14-Gn+1** and **14-Gn+2** series. The [*α*]_mol_ value divided by the number of chiral units is a constant for each series considered. In conclusion, only the number of chiral groups and their chemical environment influenced the chiroptical properties and not their placement within the dendrimer. On the contrary, the electrochemical behavior is extremely dependent on the generation considered and on the degree of burying within the dendrimer [29]. The electrochemical study of the water-soluble dendrimers **15-G3^+^** and **15-G3^−^** could not be performed, due to a poor solubility in the media used [30].

## 4. Catalysis Experiments with Ferrocenyl Phosphine Complexes on the Periphery of Dendrimers

**Figure 13:**
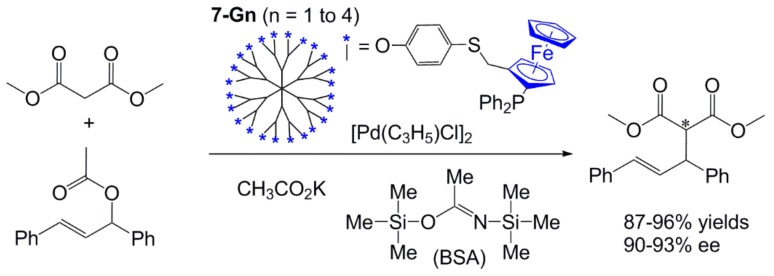
Asymmetric allylic substitution reaction, catalyzed by dendrimers **7-Gn**.

The presence of phosphines on the surface of dendrimers enabled the complexation of metals, and the study of their catalytic properties [31]. The first attempt was carried out with the chiral family **7-Gn** (**n** = 1–4), complexing [PdCl(allyl)]_2_, and used for catalyzing asymmetric allylic substitution reaction. The reaction was carried out with 1,3-diphenylprop-2-enyl acetate and dimethyl malonate, in the presence of potassium acetate and N,O-bis(trimethylsilyl)acetamide (BSA), using 2.1 mol% of phosphine (in dendrimers **7-Gn**)and 2 mol% of palladium as catalyst (Figure 13). Isolated yields were very high, and enantioselectivities were very close to the one observed for the corresponding monomeric species; no positive or negative dendritic effects [32] were observed in this reaction [25].

**Figure 14:**
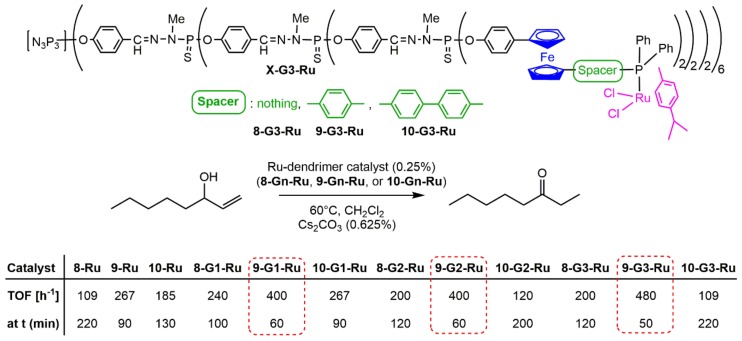
Ruthenium complexes of dendrimers **8-Gn**, **9-Gn**, and **10-Gn**, used as catalysts for the isomerization of the allylic alcohol 1-octen-3-ol to 3-octanone. Lower part: efficiency of the catalysts: TOF at 100% yield, and temperature at which it occurs.

The ruthenium complexes of the dendrimers functionalized by the ferrocenyl phosphines **8**, **9**, and **10** were used in the catalytic isomerization of the allylic alcohol 1-octen-3-ol to 3-octanone (Figure 14). Turnover frequencies (TOF: mol product/(mol catalyst × time) were calculated at the time (t) at which the yield was 100% (table in Figure 14). The dendritic catalysts clearly exhibit higher activities than their monomeric analogues, and among the three families, the **9-Gn-Ru** family is the most active [26].

**Figure 15:**
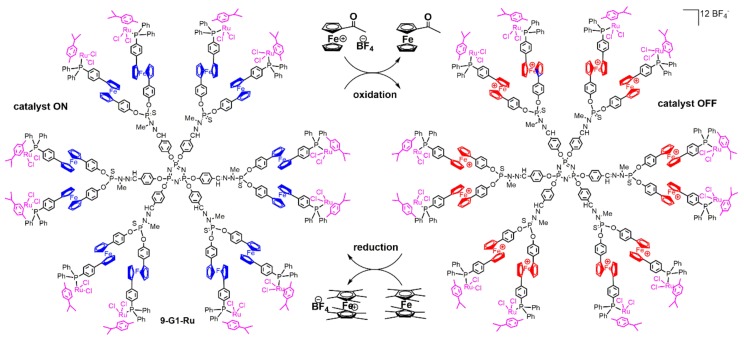
Oxidation of dendrimer **9-G1-Ru**, and then reduction of the ferrocenium dendrimer.

The most efficient family, especially the monomer (**9-Ru**) and the corresponding first generation (**9-G1-Ru**), were chosen to be used in a redox-switchable catalytic process. This was the very first example of a dendrimer used in such type of process. The catalyzed reaction was the same as in Figure 14, i.e., the isomerization of the allylic alcohol 1-octen-3-ol to 3-octanone. By adding a chemical oxidant ([Fe{η^5^-C_5_H_4_C(O)Me}Cp][BF_4_]) or reductant ([FeCp*_2_] (Cp* = C_5_Me_5_)) (Figure 15), it was possible to reversibly switch the catalytic activity of the complexes. The ferrocenium moiety obtained after oxidation withdraws electron density from the phosphine. The resulting electron-poor ruthenium center shows much lower activity, and the reaction rate is markedly reduced [33].

## 5. Conclusions

The marriage between dendrimers and ferrocene derivatives is indeed fruitful, with each component providing its specificities. The presence of ferrocenes inside the structure of dendrimers induces a detrimental influence on the electrochemical properties, as the ferrocene’s burial in the structure increases. On the contrary, the presence of ferrocenes on the surface of dendrimers provides clean electrochemical data, even for very high generations, such as the eleventh generation. The presence of a phosphine in close proximity of the ferrocene on the surface provides additional properties after the complexation with a metal, in particular for carrying catalysis experiments. Several examples have been already proposed, such as the complexation of palladium for catalyzing asymmetric allylic substitution reaction, and the complexation of ruthenium for the catalytic isomerization of the allylic alcohol 1-octen-3-ol to 3-octanone. In the latter case, the oxidation of the ferrocene induces a strong decrease of the catalytic properties, which are recovered upon reduction of the ferrocenium to ferrocene. This is the very first example of a switch ON/OFF/ON of a dendritic catalyst. Work is in progress to expand the scope of the catalytic properties of ferrocenyl dendrimers.
